# COMUS: Clinician-Oriented locus-specific MUtation detection and deposition System

**DOI:** 10.1186/1471-2164-10-S3-S35

**Published:** 2009-12-03

**Authors:** Sungwoong Jho, Byoung-Chul Kim, Ho Ghang, Ji-Han Kim, Daeui Park, Hak-Min Kim, Soo-young Jung, Ki-young Yoo, Hee-Jin Kim, Sunghoon Lee, Jong Bhak

**Affiliations:** 1 Korean Bioinformation Center (KOBIC), KRIBB, Daejeon, 305-806, Korea; 2 Korea Hemophilia Foundation Clinic 1628-26, Seocho-dong, Seocho-gu, Seoul, Korea; 3 Department of Laboratory Medicine & Genetics, Samsung Medical Center, Sungkyunkwan University School of Medicine, Seoul, 135-710, Korea; 4 University of Science & Technology (UST), Daejeon, 305-333, Korea

## Abstract

**Background:**

A disease-causing mutation refers to a heritable genetic change that is associated with a specific phenotype (disease). The detection of a mutation from a patient's sample is critical for the diagnosis, treatment, and prognosis of the disease. There are numerous databases and applications with which to archive mutation data. However, none of them have been implemented with any automated bioinformatics tools for mutation detection and analysis starting from raw data materials from patients. We present a Locus Specific mutation DB (LSDB) construction system that supports both mutation detection and deposition in one package.

**Results:**

COMUS (Clinician-Oriented locus specific MUtation detection and deposition System) is a mutation detection and deposition system for developing specific LSDBs. COMUS contains 1) a DNA sequence mutation analysis method for clinicians' mutation data identification and deposition and 2) a curation system for variation detection from clinicians' input data. To embody the COMUS system and to validate its clinical utility, we have chosen the disease hemophilia as a test database. A set of data files from bench experiments and clinical information from hemophilia patients were tested on the LSDB, KoHemGene http://www.kohemgene.org, which has proven to be a clinician-friendly interface for mutation detection and deposition.

**Conclusion:**

COMUS is a bioinformatics system for detecting and depositing new mutations from patient DNA with a clinician-friendly interface. LSDBs made using COMUS will promote the clinical utility of LSDBs. COMUS is available at http://www.comus.info.

## Background

Genetic mutations have two major types: large mutation (deletion, insertion, duplication, and inversion) and point mutation (nonsense, missense, and frame shift). Some mutations can induce DNA transcription and translation errors eventually causing protein dysfunction that leads to disease [1,2]. Currently, many whole genome scale association studies between disease and variation are being published [3]. However, medical researchers have had to go through mutations in patient DNA to detect mutations that may be the cause of a disease [4,5].

There are many human disease gene databases that contain disease-causing mutation information as locus-specific databases (LSDBs). Also, large databases, such as Online Mendelian Inheritance in Man (OMIM) [6] and the Human Gene Mutation Database (HGMD) [7], collect and describe comprehensively all disease-related genes. In contrast, LSDBs usually describe variations in a small number of genes. The LSDBs aim to provide particular genetic mutation information for disease-causing genes. The Human Genome Variation Society (HGVS) has incorporated information from many LSDBs for rare human disorders. The key activities of HGVS for LSDB construction were: 1) collecting mutations and databases by inviting reviewers of mutations, 2) creating guidelines for mutation nomenclature, 3) initiating quality control of LSDB content, and 4) specifying the minimum content of LSDBs [8].

In order to improve the mutation collection, several programs were created for an automated LSDB creation. The UMD (Universal Mutation Database) [9], LOVD (Leiden Open Variation Database) [10], MuStaR (Mutation Storage and Retrieval) [11], and MUTbase [12] are major LSDB creation programs and resulting databases. Curators wishing to construct an LSDB use the programs according to their specific disease targets. However, these programs do not support any bioinformatics sequence analysis method for variation deposition.

Generally, variation detection is achieved with sequencing patient DNA, the key activity for variation detection. However, clinicians who study disease-causing mutation are usually not experts on analyzing sequences. In order to encourage their data submission to LSDBs, simpler and more convenient program interface is necessary. We have developed a simple LSDB construction system that supports mutation detection and deposition to promote easier mutation data submission and maintenance http://www.comus.info.

As a test database, we built a hemophilia disease LSDB. The disease hemophilia (hemophilia A, HA [MIM #306700]; hemophilia B, HB [#306900]) is one of the most historical and archetypical Mendelian disorders in human. Patients with hemophilia suffer from uncontrolled bleeding from factor VIII or IX deficiency due to a mutation in either the F8 (HA) or F9 (HB) gene, respectively, on Xq27.1~q28. The clinical utility of COMUS was validated in this test LSDB, called KoHemGene http://www.kohemgene.org, using a set of raw data files from direct sequencing, as well as clinical information from hemophilia patients.

## Methods

Our system consists of a database and web application. The web application was constructed for mutation candidate prediction, submission, and registration. Our system was constructed using the JSP programming language and MySQL database.

### Database structure

**Figure 1:**
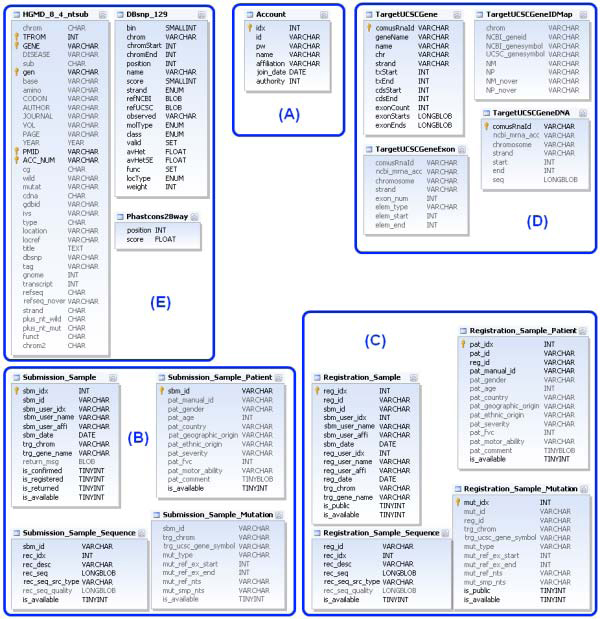
**Database structure**. Database tables are classified into five parts: (A) account, (B) submitted data, (C) registered data, (D) gene information, and (E) known mutation data from public databases (HGMD, dbSNP).

The COMUS database tables are classified into five parts: account, submitted data, registered data, gene information, and known mutation data from public databases (HGMD, dbSNP) (Figure 1). For submitted and registered data, submission IDs and registration IDs are assigned as reference keys to integrate their related tables. The gene information part contains gene structure information, UCSC evolutionary conservation score, and the external gene ID links. The mutation data part contains known mutation information from HGMD and dbSNP. This part is used to define novel mutations from patients.

### Predicting mutation candidate

The user can submit data using an AB1 file (chromatogram files used by instruments from Applied Biosystems) or a FASTA file as input. When the user inputs an AB1 file, the web application checks the quality of the sequence and converts it to FASTA format using the Phred program [13,14]. After preparing FASTA sequence files, the sequences are aligned to reference sequences of the gene locus using the BLAT program [15]. In order to compare patient data with known sequences, we extracted the genome sequence and gene structure information from UCSC Hg18 [16]. Input patient sequences are then aligned to reference genomic regions. After that, we calculate various mutation types, such as mismatch, insertion, and deletion, as mutation candidates. In order to define novel mutations, we compare the genomic positions of mutation candidates to known variations from public databases. To explore the evolutionary constraints of mutation candidates, we calculate evolutionary conservation scores using those UCSC phastCons score [17]. Finally, amino acid changes caused by each mutation candidate are analyzed.

### Submission interface

**Figure 2:**
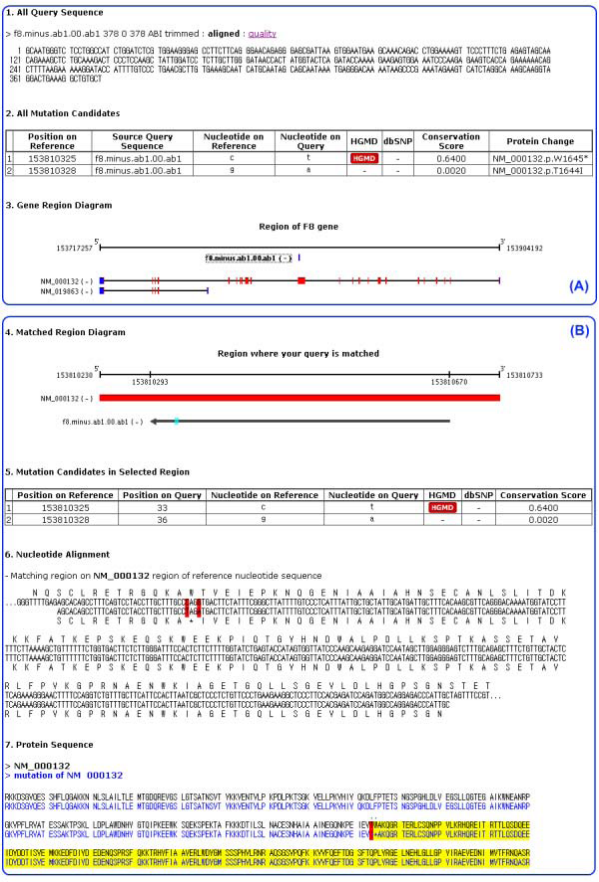
**Report from the mutation candidate prediction system**. (A) After query sequence analysis, this mutation candidate prediction system reports all query sequences, all mutation candidates, and diagrams of target gene locus with query sequence. In the gene region diagram, the top line represents a gene locus of a target gene, and the blue bar below the top line represents a mapped query sequence. The next two lines represent mRNAs in the target gene locus. The blue box is UTR regions, and the red box is coding regions. (B) The detailed information of selected query sequence is shown. The top line represents a gene locus of a target gene, the red bar represents coding region, the black arrow represents the mapped query sequence, and cyan boxes are mutation candidates. The mutation candidate table shows mutation type, chromosome position, and the existence of the mutation candidate in public databases. Nucleotide alignment and protein sequences of the query sequence are shown.

The user can analyze patients' sequences using several additional options. COMUS accepts input files in AB1 and FASTA formats. When a submitter checks the AB1, a trimming cut-off bar is activated for quality control. Generally, in order to increase the sequence accuracy, the user does multiple sequencing runs for the patients' genomic regions. Therefore, our system is constructed to support multiple input sequences, and then multiple alignments are used for predicting mutation candidates. After the mutation candidate prediction process, the user can see input sequences, a mutation candidate list, and an input sequence mapping image against reference gene DNA sequences. If the user clicks input sequences in the mapping image, then the detailed alignment information, a mutation candidate list on the clicked input sequences, and protein sequences of the input sequences compared to protein sequences from the gene can be seen (Figure 2). After the mutation candidate prediction, the user can select a mutation(s) and add patient private information for submission.

### Curator interface

In order to curate submitted data, we created a curator account in the web application. Curators can approve any user's account (submitter account) and can see all the submitted sequence data. When the curators register submitted data, they can use the mutation predicting system to check whether the submitted mutations are appropriate to deposit into their target LSDB. If the submission is not appropriate, curators can return the submission with a return message. With a curator's approval, the submitted sequences and mutation are deposited into LSDB.

## Results and discussion

We have constructed a mutation candidate detection and deposition system, COMUS. COMUS addresses two disadvantages in common LSDB systems such as LOVD and UMD. First, we incorporated a mutation prediction system which supports clinicians' mutation data identification and submission. Second, COMUS alleviated the time-consuming bottleneck of specialized curators maintaining the LSDB systems. Because COMUS makes it possible to work with an integrated mutation prediction system, anyone, especially the major variation detectors who are often clinicians or field workers, can be curators. To construct a useful LSDB, some private patient information is necessary. However, because the specifics of patient information vary depending on the disease, COMUS supports only fundamental specifics among patient information: patient ID, gender, age, country, geographic origin, ethnic origin, disease severity, forced vital capacity (FVC), motor ability, and comments.

**Figure 3:**
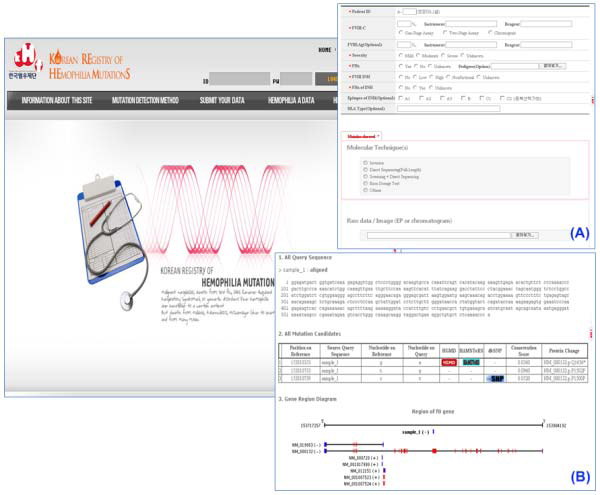
**Korean registry of hemophilia mutations (KoHemGene)**. (A) The KoHemGene system collects patient information using this submission page. (B) The mutation candidate prediction system on the KoHemGene site came from COMUS.

Using the COMUS system, an LSDB for hemophilia (KoHemGene) was constructed http://www.kohemgene.org (Figure 3). Using the KoHemGene database, clinicians can directly upload raw data files obtained from sequencing experiments to detect any sequence variations. Any variation detected is described according to the HGVS (Human Gene Variation Society) guidelines and analyzed with reference to relevant databases, including HGMD. Clinicians can also input clinical data to link them with genetic information to get genotype-phenotype correlations.

Recently, Next Generation Sequencing (NGS) technologies are quickly developing and several complete human genomes have been sequenced with NGS. However, in clinics, NGS is not widely used due to difficulty of processing enormous amounts of data, and the current high cost of NGS. As Sanger sequencing machines are still the main facilities in clinics or small-scale wet-labs, COMUS was constructed focusing on Sanger sequencing data as input. When the cost of NGS decreases in the near future, clinicians will use NGS as the method of patient mutation detection.

## Conclusion

COMUS is a comprehensive bioinformatics system that has been developed to efficiently bridge genetic data from benchwork to clinics and bedsides. Tailored to have a clinician-friendly interface, COMUS is believed to promote the clinical utility of LSDBs and thereby facilitate translational research in the field of medical genetics, particularly in terms of genotype-phenotype correlations.

## Competing interests

The authors declare that they have no competing interests.

## Authors' contributions

SJ and KJH created the web application of COMUS. SJ designed the database. BK and HMK supported the database design. SJ, BK, and HG initiated this project and wrote the manuscript. DP assisted in the manuscript writing. KYY provided patient samples and clinical information and designed the information contents of KoHemGene. HJK provided genetic data of hemophilia patients and designed the information contents of KoHemGene. SYJ, SL, and JB directed the study.

## Note

Other papers from the meeting have been published as part of *BMC Bioinformatics *Volume 10 Supplement 15, 2009: Eighth International Conference on Bioinformatics (InCoB2009): Bioinformatics, available online at http://www.biomedcentral.com/1471-2105/10?issue=S15.
